# From Schooling to Shoaling: Patterns of Collective Motion in Zebrafish (*Danio rerio*)

**DOI:** 10.1371/journal.pone.0048865

**Published:** 2012-11-14

**Authors:** Noam Miller, Robert Gerlai

**Affiliations:** 1 Department of Ecology and Evolutionary Biology, Princeton University, Princeton, New Jersey, United States of America; 2 Department of Psychology, University of Toronto at Mississauga, Mississauga, Ontario, Canada; Cajal Institute, Consejo Superior de Investigaciones Científicas, Spain

## Abstract

Animal groups on the move can take different configurations. For example, groups of fish can either be ‘shoals’ or ‘schools’: shoals are simply aggregations of individuals; schools are shoals exhibiting polarized, synchronized motion. Here we demonstrate that polarization distributions of groups of zebrafish (*Danio rerio*) are bimodal, showing two distinct modes of collective motion corresponding to the definitions of shoaling and schooling. Other features of the group's motion also vary consistently between the two modes: zebrafish schools are faster and less dense than zebrafish shoals. Habituation to an environment can also alter the proportion of time zebrafish groups spend schooling or shoaling. Models of collective motion suggest that the degree and stability of group polarization increases with the group's density. Examining zebrafish groups of different sizes from 5 to 50, we show that larger groups are less polarized than smaller groups. Decreased fearfulness in larger groups may function similarly to habituation, causing them to spend more time shoaling than schooling, contrary to most models' predictions.

## Introduction

Living and traveling in groups confer multiple benefits in avoiding predation and improved foraging [1] and individuals of a majority of fish species spend some part of the their lives in groups [2]. Groups of fish are commonly termed either shoals or schools and several authors have drawn a distinction between the two terms [3]: shoal refers to any group of fish that “remain together for social reasons” (i.e., not solely due to an external stimulus [4]) whilst schools are shoals that are “polarized and coordinated” [4]. Polarization is a measure of the degree to which members of the group are moving in the same direction. Being in a polarized group may confer anti-predatory advantages beyond those available in a disordered shoal, for instance through increased predator confusion [5], and may make it easier to detect any sudden deviation in the heading of a conspecific, potentially a sign of impending danger [1]. Polarized motion may also help fast-moving groups to stay together (or even be required to maintain the cohesion of fast groups) – particularly important for migrating species – and may improve the flow of information through the group [6], [7].

Additionally, some theoretical models of collective motion predict a sharp density-dependent transition from a low polarization regime (corresponding to shoaling) to a high polarization regime (schooling [8]). However, these models do not consider indirect effects on collective motion resulting from changes in group size. For instance, members of larger groups may experience lower stress levels than members of smaller groups and these changes may themselves affect the polarization of the group.

Despite broad acceptance of the distinction between schooling and shoaling [4], no work that we are aware of has examined the empirical characteristics of each behavioral mode (as called for, e.g., by [9]) or sought to find what determines which of the two modes of motion groups adopt, other than group density. Here we show that polarization distributions of groups of zebrafish (*Danio rerio*) are bimodal, implying a behavioural distinction between highly polarized groups – schools – and weakly polarized groups – shoals – and that other features of collective motion, such as the mean speeds of group members and the mean spacing between them, correlate with polarization (Experiment 1). We also show that the dominant mode of motion exhibited by a group depends on several environmental conditions such as the group's habituation to the environment (Experiments 1 and 2) and the size of the group (Experiment 3). Interestingly, in our data polarization is negatively correlated with the size of the group, in contrast to the predictions of some models. We suggest that this may result from a failure of these models to consider indirect effects of changes in group size.

## Results

### Experiments 1 and 2

**Figure 1:**
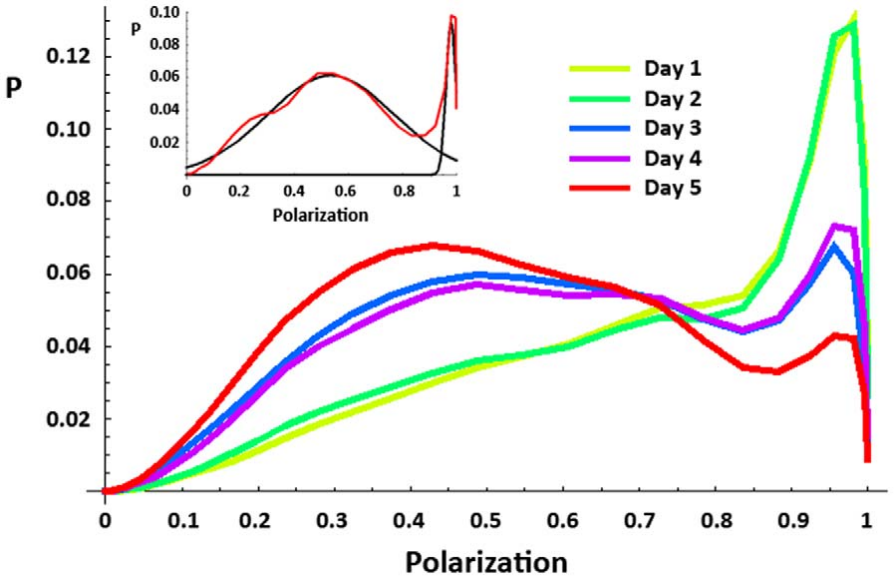
Density distributions of polarization by testing day for Experiment 1. Summed polarization distributions for repeated exposures to the testing tank on consecutive days. Distributions are averaged across the entire session (1800 frames per session) and over all groups (24 groups of 8 fish each). As fish habituate to the tank across days they spend more time shoaling (low polarization) and less time schooling (high polarization; K-S test, days 1–2 vs. days 3–5, all p<0.0001; see Table S1). The inset shows a sample distribution from one complete session (1800 frames) (red) and its decomposition into schooling and shoaling modes (black), by fitting a Gaussian mixed model (see text). Note that each session's distribution was tested for bimodality independently.

Using detailed swimming trajectories of groups of 8 zebrafish each (see Methods), we constructed the mean distributions of the polarization of the group. Polarization distributions were bimodal (Figure 1), implying that groups of zebrafish often form either highly polarized schools or weakly polarized shoals but take intermediate forms less frequently. We explicitly tested each distribution for bimodality (see Methods): of 118 analyzed distributions, all but 21 were bimodal and the peaks of most distributions that were unimodal fell clearly into one or the other behavioral form (Figure S2).

**Figure 2:**
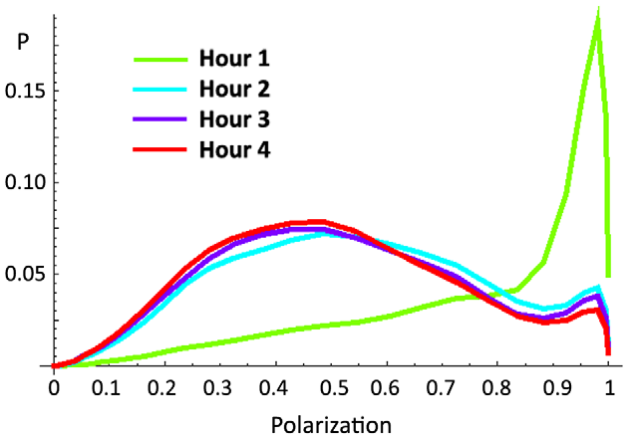
Density distributions of polarization by hour for Experiment 2. Summed polarization distributions for each hour of a single, 4 hour, exposure to the testing tank. Distributions are averaged across the entire session (1800 frames per session) and all groups (8 groups of 8 fish each). A similar effect of habituation is seen to that observed in Figure 1, with increased shoaling and decreased schooling as time passes (K-S test, hour 1 vs. hours 2–4, all p<0.001; see Table S2).

**Figure 3:**
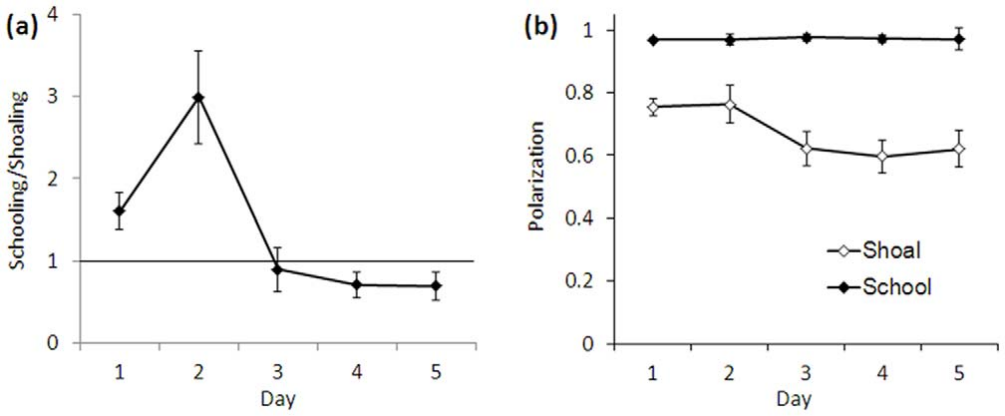
Ratio of the mean time spent schooling to shoaling (a) and mean modes of polarization distribution components (b) by testing day in Experiment 1. Unimodal data distributions were excluded. Error bars represent ± SEM. The spike in time spent schooling on day 2 (a) is attributable to a few outlier sessions.

Polarization distributions changed dramatically with repeated exposure to the testing tank (Figure 1; Table S1), becoming increasingly biased towards shoaling. A similar effect was observed across multiple hours of a single extended exposure to the testing tank (Figure 2) and these effects were highly significant (Tables S1, S2). In other words, zebrafish groups spent more time shoaling and less time schooling as they habituated to the testing environment (Figure 3a). The degree of polarization of schools (the position of the peak corresponding to schooling in the distribution) did not change across repeated exposures to the tank (Repeated Measures ANOVA, F(4, 24) = 1.994, p = 0.128) but the degree of polarization of shoals decreased, i.e., shoals became more disorganized (F(4,24) = 2.816, p = 0.048) as fish habituated to the tank (Figure 3b). This result implies that schooling is a fixed behavioral pattern whereas the characteristics of shoals may depend on environmental factors (such as habituation).

Next, we segmented all the trajectories by which mode they fell into: each frame of data was designated either a schooling or shoaling frame by the polarization of the group at that time-point (unimodal distributions were excluded from this analysis). Most segments lasted only a few seconds, reflecting rapid changes in the groups' polarization, but groups spent over a third of their time (36.4%) in schooling or shoaling segments lasting longer than 30 sec. 2.45% of schooling and 8.87% of shoaling segments lasted for more than 1 min. Shoaling segments were longer, on average, than schooling segments (Figure S3; Table S3).

**Figure 4:**
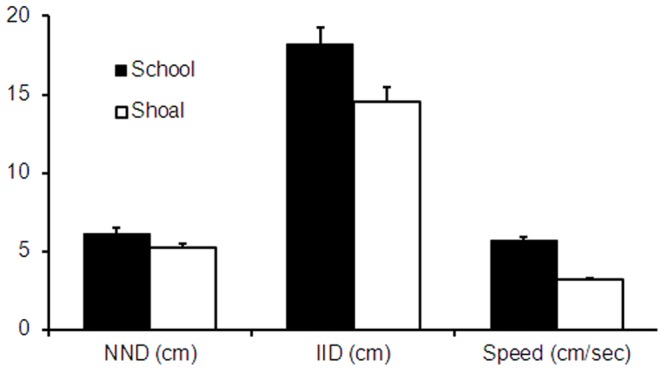
Differences in Nearest Neighbor Distance (NND), Inter-Individual Distance (IID), and mean speed between shoals and schools in Experiment 1 (means of 24 groups of 8 fish each). All differences were significant (paired-sample t-test, all p<0.0001). Error bars represent ± SEM.

Finally, we compared the two modes of behaviour on other measures of collective motion: for each frame in each behavioral mode we measured the mean Nearest Neighbor Distance (NND), mean Inter-Individual Distance (IID; the mean distance between an individual and all other members of the group), and mean individual speed (Figure 4). Fish were further apart when in schools than when in shoals (paired-sample t-test; NND, t = 8.51, p<0.0001; IID, t = 9.59, p<0.0001) and swam faster (t = 14.92, p<0.0001). The latter distinction, a positive correlation between speed and polarization, has been previously noted in other fish species [10].

### Experiment 3

**Figure 5:**
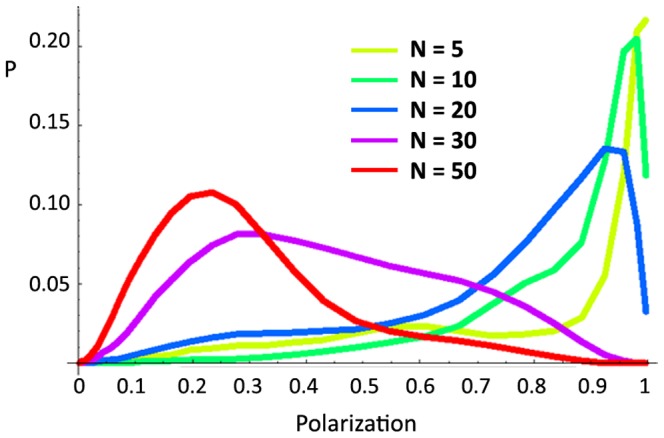
Density distributions of polarization by group size for Experiment 3. Summed polarization distributions for each group size (N = 5, 10, 20, 30, and 50). Distributions are averaged across the entire session (1800 frames per session) and all groups (4 groups of each size). Larger groups are significantly less polarized than smaller groups (K-S test, all p<0.0001; see Table S3).

We next tested how changes in group size affect which behavioral mode the group inhabits. Some models of collective motion [8] suggest that the polarization of a group depends on its size or density, an effect that has also been observed in some behavioral data [11], [12]. In these situations, both theoretical and empirical, small groups have widely and rapidly varying polarization; as group size increases polarization both increases and becomes more stable, so that large groups are highly polarized and rarely change direction. We tested groups of zebrafish over a range of group sizes (N = 5, 10, 20, 30, and 50) and applied the same analyses to the data as above (sample polarization time-series' are presented in Figure S4). Figure 5 shows the summed polarization distributions for all group sizes. Contrary to our expectations, and the predictions of the models, larger zebrafish groups were significantly less polarized, on average, than smaller groups (Table S4), spending more of their time shoaling and less time schooling. NND did not vary consistently across group sizes (Figure S5A). Larger shoals were somewhat slower on average than smaller shoals (Figure S5B), though this may simply reflect the greater amount of time they spent shoaling. Note that the mean NND of groups in this experiment was lower than that of groups in Experiment 1.

## Discussion

Above, we show that zebrafish more frequently form either highly or weakly polarized groups and rarely take intermediate forms. We call these modes schooling and shoaling, respectively, following the terminology common in the literature [4]. However, it might be argued that the distinction between schools and shoals should refer only to changes that persist for longer periods of time and that the fluctuations in polarization in our data, often lasting only a few seconds (Figure S3), represent no more than noise around one mode or the other. Nonetheless, our data demonstrate that groups of zebrafish exist predominantly in two statistically distinct modes of collective motion rather than intermediate forms. We also show that both social environment and internal states can affect the distribution of group polarization which, in some cases, might lead to long periods spent strictly in one mode or the other. Such situations would more closely correspond to the traditional distinction between schooling and shoaling but would, according to this account, represent merely extreme manifestations of the same mechanisms revealed in our data.

Additionally, the walls of the testing tank might have had different effects on large vs. small or on habituated vs. stressed groups. Zebrafish (and other species) tend to remain close to the walls of novel environments and this thigmotactic response might have acted to linearize, and thus polarize, the fish in our groups. Larger (more numerous) groups may have filled a larger portion of the tank and thus been unable to remain sufficiently close to the walls of the tank for any polarizing effect to be felt throughout the group. However, we note that in our data, large and habituated groups were still able to achieve high levels of polarization, equal to those demonstrated by smaller or more stressed groups, they simply did so less often (or for shorter periods of time). Thus, as shown in Figure 1, habituating and habituated groups still displayed schooling behavior which was as polarized as their schooling on earlier days (Figure 3B) but they performed this behavior less often.

Partridge [9] suggested that the collective motion of different species of fish might best be characterized by the proportion of time that they spend schooling. Our data (e.g., Figure 2B) demonstrate that factors other than species also contribute to this ratio. Both group size and habituation to the environment affect the proportion of time zebrafish groups spend in each mode. Rather than smoothing out the polarization distribution and eliminating its bimodality, changes in these variables serve primarily to alter the relative amplitudes of the two modes, suggesting that they mark two distinct methods of forming a group. This suggestion is supported by differences in other characteristics, such as the speed and density of the group, between the two modes.

The changes we observed in polarization and in the proportion of time spent in each of the two modes of motion may be explained by changes in the level of fear (or stress) of the fish. As fish habituate to the testing environment, either over repeated exposures to it (Experiment 1) or simply over time during a single exposure (Experiment 2), or when fish are in a larger group (Experiment 3), they might be less fearful and therefore more likely to adopt a shoaling configuration rather than schooling. This would further suggest that schooling behaviour is maintained, under stressful conditions, at some energetic or cognitive cost over shoaling behaviour. This cost might be associated with greater attention required to school [5] or, indirectly, with the metabolic costs of swimming faster (as we and others have shown that individuals in schools swim more quickly than those in shoals).

Schooling may provide greater protection from predation than shoaling but may hinder individual foraging, particularly for individuals near the back of the school. We [13] have previously shown that the density of zebrafish groups oscillates with a characteristic frequency and suggested that this may represent a solution to the trade-off between protection, which is enhanced by closeness to conspecifics, and foraging, which is easier at some distance from the rest of the group [14]. The present data demonstrate another way that zebrafish balance these conflicting requirements, by adjusting the proportion of time they spend shoaling or schooling to match their current social conditions.

Most models of collective motion assign a set of simple rules to each individual, dependent on its local environment [8], [15]–[22] and the combination of many such local interactions gives rise to globally coordinated motion, closely simulating the movement of real groups of fish. Many of these models exhibit both shoaling and schooling under different parameter regimes (e.g., [15]). In some cases increasing the size of the simulated group, and thus the number of local interactions, leads to an increase in polarization and stabilization of the polarization [8], [17], a result that is supported by some empirical data [11], [12]. In our data, larger groups of zebrafish were more likely to shoal, displaying low polarization, than smaller groups, contrary to the predictions of such models. This may reflect an additional behavioral ‘rule’ that is not accounted for by current models of collective motion and plays a smaller role in the species studied to date under varying densities [11], [12], possibly one that depends on the stress or fear levels of the animals, as suggested above. This effect, in our data, is sufficiently powerful to completely overshadow any increase in polarization resulting from larger group sizes. Recently, a possible mechanism has been suggested [5] by which an increase in stress levels may lead to individuals increasing the rate at which they examine their relative position and orientation to the group and this increased ‘update rate’ may result in increased polarization.

Our data provide quantitative support for the long-standing definition in the literature between schooling and shoaling and demonstrate how some environmental variables can affect the proportion of time zebrafish groups spend in each mode. To the best of our knowledge, this distinction has been studied only in groups of fish. However, it is likely that similar processes of habituation and similar group-size effects to those observed here operate in other group-living taxa. Do herds of mammals or flocks of birds also have two (or more) distinct modes of collective motion? Examining the polarization distributions of other species may reveal interesting similarities and differences in their methods of collective motion.

## Methods

### Ethics statement

All animal procedures were approved by the University of Toronto at Mississauga Animal Care Committee and followed the guidelines set by the Canadian Council on Animal Care (CCAC).

### Subjects

Subjects were all laboratory-reared sexually mature zebrafish 3–12 months of age and were 3–4 cm long. All fish were bred in-house. In Experiment 1, 24 groups of 8 fish per group were tested for five consecutive days each; In Experiment 2, 8 groups of 8 fish each were tested once each; and in Experiment 3, 4 groups of each size (N = 5, 10, 20, 30, and 50 fish) were tested once each. All groups consisted of approximately equal numbers of males and females. Groups of fish to be tested together were housed together for at least one week prior to the start of each experiment. Fish were housed in 40-litre tanks containing ‘system’ water that was previously reverse osmosis purified and mixed with sea salt (‘Instant Ocean’ sea salt, Aquarium Systems Inc., OH) so that the conductivity of the water was between 900–1200 micro Siemens (576–768 TDS ppm). The water in the tanks was filtered (Aqueon PowerFilter 30, Franklin, WI), aerated, and maintained at a temperature of 26±2°C. Lights in the room in which the fish were kept turned on at 7:00 h and off at 21:00 h. Fish were fed flake food (Tetramin Tropical Flakes, Tetra, USA) *ad lib*, 30 min before each testing session.

### Apparatus

Fish were tested in a circular white plastic tank with a diameter of 91 cm, filled with system water to a depth of 10 cm (Figure S6), located in one corner of the room where they were housed. The temperature of the water in the tank was kept at 26±2°C. A moveable black room divider visually separated the tank from the rest of the room. The tank was lit by two fluorescent light fixtures placed at opposite sides of the tank, ensuring an even level of illumination in all parts of the tank. Experiments were filmed using a Sony Handycam (model HDR-XR-520) attached to the ceiling with the lens of the camera 200 cm above the surface of the water, located so that the entire tank was visible in the frame of the video. Videos, at 1920×1080 pixels and 12 fps, were converted into AVI format using iSkySoft Video Converter (v 2.2).

### Procedure

All testing was performed between 10:00 and 14:00 h each day. Fish were gently netted from their home tank and transferred in a plastic beaker to the testing tank. They were then gently poured into the center of the tank. After the session ended, fish were netted into the beaker and returned to their home tank. Sessions lasted for 30 min in Experiments 1 and 3, and for 4 hours in Experiment 2. The water in the testing tank was replaced after each two groups had been tested to control for possible odor cues left by previous groups. Sample videos of the sessions are in the Supplementary Materials (Videos S1 S2, S3, S4, S5, S6, S7, S8, S9, S10).

### Data analysis

A custom application (described in [23]) extracted the swim path trajectories of all the fish for a 5 min segment of each trial, starting 5 min after the fish were placed in the testing tank. Briefly, the tracking system identified each fish in each frame of video by subtracting it from a reference image that did not contain the fish and locating ‘clumps’ of pixels that were sufficiently different in the two images. Locations were then stitched together into trajectories assuming that fish did not move very far in the time between consecutive frames (0.08 sec). Occlusions (when two or more fish occupied the same clump) were resolved by assuming that fish turned minimally during the occlusion or, when necessary, manually (see [23] for details). In Experiments 1 and 3 only one 5 min segment per session was coded; in Experiment 2, one 5 min segment was coded for each hour of the session (i.e., mins 5–10, 65–70, 125–130, and 185–190). Raw trajectories were analyzed using *Mathematica* (v 7.0; Wolfram Research). The trajectories were smoothed with a linear (unweighted) moving average, using a window of 0.5 sec. Any fish that had temporarily left the group, as defined in [24], were excluded from analysis for the duration of their excursion. Individuals or sub-groups of size *n* were considered to be separate from the main shoal (on an excursion) when their NND_n_ (i.e., the distance to their *n*th nearest neighbor) was greater than the mode of the distribution of all NND_n_ (see [24] for details). Using the data from Experiment 3 we demonstrate, however, that considering only cohesive groups did not change the basic features of the resulting distributions (Figure S1b). However, the data with excursions removed are presented in the main text as this method more accurately reflects the behavior of the shoal [24].

Polarization was defined in 2 different ways, to control for possible boundary effects caused by the curved wall of the testing arena. The data presented in the main text use the standard definition of polarization as the magnitude of the mean movement vector of all members of the group (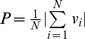, where *v_i_* is the movement vector of fish *i* in a group of size N). However, large groups swimming along the walls of our circular tank might be less polarized than smaller groups simply because they adhere to the curvature of the walls and inhabit a larger arc of the circle. To overcome this difficulty, we recalculated the orientation of each fish in each frame relative to the (tangent to the) wall of the tank at the nearest point to the fish, effectively linearizing the tank walls. We then recalculated the polarization of the group using these relative orientations. We show that the polarization distributions generated using this modified procedure are not qualitatively different from those generated by the standard measure (Figure S1a).

Polarization distributions were individually tested (i.e., the distribution from each session was tested) for bimodality using a Maximum Likelihood Estimation (MLE) method: the best-fit mixed-Gaussian models with one, two, and three components were found for each distribution; likelihood scores were calculated for each model; and the Bayesian Information Criterion (BIC) was used to select the most likely model [25]. For bimodal distributions, pairs of Gaussian distributions were fit to the distribution and the point at which they crossed was used as a threshold to partition the data into its component modes (Figure 1, inset). Note that, as a result, the assignment of frames of data as either schooling or shoaling is session-dependent. All statistical analyses were conducted either in *Mathematica* or SPSS (v 17.0). A significance level of 0.05 was used for all tests. In Experiment 1, 2 sessions had to be excluded from analysis due to video recording errors.

## Supporting Information

Figure S1 **Polarization distributions for Experiment 3, using alternate measures of polarization.** These figures display the same data as Figure 5 in the main text, but calculated using an alternate measure of polarization that eliminates potential effects of the curved testing tank walls (a) and without excluding fish that had left the group on excursions (b). All comparisons between the distributions for different group sizes were qualitatively identical to those for the original data, presented in the main text.
(TIF)

Figure S2 **Binned modes of unimodal polarization distributions in Experiment 1.** The largest number of unimodal distributions occurred on the first day of exposure to the testing environment (8 of 21) and represent sessions in which the group spent most of its time schooling (high polarization).
(TIF)

Figure S3 **Survival curves for schooling (a) and shoaling (b) segments for Experiment 1.** The curves show the proportion of segments of each mode that lasted longer than a given length of time. Mean schooling segment length was 4.7 sec and mean shoaling segment length was 9.4 sec. Data are plotted on a Log-linear axis.
(TIF)

Figure S4 **Sample time-series' of polarization.** Each panel shows the polarization of a single group for the first 2 min of a sample session. The top left panel shows a transition from schooling (high polarization) to shoaling (low polarization) from Experiment 1 (N = 8). The other 5 panels show representative samples of the polarizations of groups of different sizes (N = 5, 10, 20, 30, and 50) from Experiment 3.
(TIF)

Figure S5 **Mean NND (A) and mean speed (B) of zebrafish shoals of different sizes, from Experiment 3.** The charts show the mean NND and mean speed over all frames of all groups at each shoal size. Error bars represent ± SEM.
(TIF)

Figure S6 **Diagram of the experimental setup.** The camera was mounted on the ceiling such that the entire testing tank was visible in the frame. Two fluorescent lamps lit the tank from the sides, placed just above the lip of the tank to avoid glare in the video image. The tank was 91 cm in diameter and filled with water to a depth of 10 cm. Adapted from Miller, N. & Gerlai, R. (2012). Automated tracking of zebrafish shoals and the analysis of shoaling behavior. In A.V. Kalueff and A.M. Stewart (eds.) *Zebrafish protocols for neurobehavioral research* (New York: Humana Press) pp. 217–230.
(TIF)

Table S1 **Comparisons of polarization distributions by day in Experiment 1.** Summed distributions (shown in Figure 1) were compared between repeated exposures to the testing environment using a 2-sample Kolmogorov-Smirnov test. The top half of the table presents the test statistic values; the bottom half presents p-values. Non-significant p-values are shaded.
(PDF)

Table S2 **Comparisons of polarization distributions by hour in Experiment 2.** Summed distributions (shown in Figure 2) were compared between hours of the session using a 2-sample Kolmogorov-Smirnov test. The top half of the table presents the test statistic values; the bottom half presents p-values. Non-significant p-values are shaded.
(PDF)

Table S3 **Schooling and shoaling segment lengths by day in Experiment 1.** Mean durations of schooling and shoaling segments for each day of Experiment 1. Mean segment lengths ± standard deviations are given in seconds.
(PDF)

Table S4 **Comparisons of polarization distributions by group size in Experiment 3.** Summed distributions (shown in Figure 5) were compared using a 2-sample Kolmogorov-Smirnov test. The top half of the table presents the test statistic values; the bottom half presents p-values.
(PDF)

Video S1 **Sample video from Day 1 of Experiment 1.**
(AVI)

Video S2 **Sample video from Day 2 of Experiment 1, of the same individuals as Video S1.**
(AVI)

Video S3 **Sample video from Day 3 of Experiment 1, of the same individuals as Video S1.**
(AVI)

Video S4 **Sample video from Day 4 of Experiment 1, of the same individuals as Video S1.**
(AVI)

Video S5 **Sample video from Day 5 of Experiment 1, of the same individuals as Video S1.**
(AVI)

Video S6 **Sample video from Experiment 3, of a group of 5 fish.**
(AVI)

Video S7 **Sample video from Experiment 3, of a group of 10 fish.**
(AVI)

Video S8 **Sample video from Experiment 3, of a group of 20 fish.**
(AVI)

Video S9 **Sample video from Experiment 3, of a group of 30 fish.**
(AVI)

Video S10 **Sample video from Experiment 3, of a group of 50 fish.**
(AVI)

## References

[1] Krause J, Ruxton GD (2002) Living in Groups. Oxford: Oxford University Press. 228 p.

[2] Shaw E (1978) Schooling fishes. Am Sci 66: 166–175.

[3] Pitcher TJ (1983) Heuristic definitions of fish shoaling behaviour. Anim Behav 31: 611–613.

[4] Pitcher TJ, Parrish JK (1993) Functions of shoaling behavior in teleosts. In: Pitcher TJ, editor. Behaviour of Teleost Fishes. London: Chapman & Hall. pp. 363–439.

[5] Bode NWF, Faria JJ, Franks DW, Krause J, Wood JA (2010) How perceived threat increases synchronization in collectively moving animal groups. Proc R Soc B 277: 3065–3070. 10.1098/rspb.2010.0855 PMC2982070 20504810

[6] Couzin ID, Krause J, Franks NR, Levin SA (2005) Effective leadership and decision-making in animal groups on the move. Nature 433: 513–516. 15690039 10.1038/nature03236

[7] Day RL, Macdonald T, Brown C, Laland KN, Reader SM (2001) Interactions between shoal size and conformity in guppy social foraging. Anim Behav 62: 917–925.

[8] Viczek T, Czirok A, Ben-Jacob E, Cohen I, Shochet O (1995) Novel type of phase transition in a system of self-driven particles. Phys Rev Lett 75: 1226–1229. 10060237 10.1103/PhysRevLett.75.1226

[9] Partridge BL (1982) Rigid definitions of schooling behaviour are inadequate. Anim Behav 30: 298–299.

[10] Parrish JK, Viscido SV, Grünbaum D (2002) Self-organized fish schools: an examination of emergent properties. Biol Bull 202: 296–305. 12087003 10.2307/1543482

[11] Buhl J, Sumpter DJT, Couzin ID, Hale JJ, Despland E, Miller ER, Simpson SJ (2006) From disorder to order in marching locusts. Science 312: 1402–1406. 16741126 10.1126/science.1125142

[12] Becco Ch, Vandewalle N, Delcourt J, Poncin P (2006) Experimental evidences of a structural and dynamical transition in fish school. Phys A 367: 487–493.

[13] Miller N, Gerlai R (2008) Oscillations in shoal cohesion in zebrafish (*Danio rerio*). Behav Brain Res 193: 148–151. 18573546 10.1016/j.bbr.2008.05.004 PMC2709827

[14] Miller N, Gerlai R (2007) Quantification of shoaling behavior in zebrafish (*Danio rerio*). Behav Brain Res 184: 157–166. 17707522 10.1016/j.bbr.2007.07.007

[15] Couzin ID, Krause J, James R, Ruxton GD, Franks NR (2002) Collective memory and spatial sorting in animal groups. J Theor Biol 218: 1–11. 12297066 10.1006/jtbi.2002.3065

[16] Couzin ID, Krause J, Franks NR, Levin SA (2005) Effective leadership and decision-making in animal groups on the move. Nature 433: 513–516. 15690039 10.1038/nature03236

[17] Czirok A, Vicsek T (2000) Collective behavior of interacting self-propelled particles. Physica A 281: 17–29.

[18] Viscido SV, Parrish JK, Grunbaum D (2004) Individual behavior and emergent properties of fish schools: a comparison of observation and theory. Mar Ecol Prog Ser 273: 239–249.

[19] Mirabet V, Auger P, Lett C (2007) Spatial structures in simulations of animal grouping. Ecol Model 201: 468–476.

[20] Lukeman R, Li Y-X, Edelstein-Keshet L (2010) Inferring individual rules from collective behavior. Proc Natl Acad Sci USA 107: 12576–12580. 20616032 10.1073/pnas.1001763107 PMC2906562

[21] Parrish JK, Viscido SV, Grunbaum D (2002) Self-organized fish schools: an examination of emergent properties. Biol Bull 202: 296–305. 12087003 10.2307/1543482

[22] Hemelrijk CK, Hildenbrandt H (2008) Self-organized shape and frontal density of fish schools. Ethology 114: 245–254.

[23] Miller N, Gerlai R (2012) Automated tracking of zebrafish shoals and the analysis of shoaling behavior. In: Kalueff AV, Stewart AM, editors. Zebrafish Protocols for Neurobehavioral Research. New York: Humana Press. pp. 217–230.

[24] Miller N, Gerlai R (2011) Redefining membership in animal groups. Behav Res 43: 964–970. 10.3758/s13428-011-0090-z 21491173

[25] Drai D, Benjamini Y, Golani I (2000) Statistical discrimination of natural modes of motion in rat exploratory behavior. J Neurosci Meth 96: 119–131. 10.1016/s0165-0270(99)00194-6 10720676

