# Supplementary figures and images for: From Schooling to Shoaling: Patterns of Collective Motion in Zebrafish (Danio rerio)
Source: PLoS One. 2012 Nov 14;7(11):e48865. doi: 10.1371/journal.pone.0048865 (PMC3498229; doi:10.1371/journal.pone.0048865)

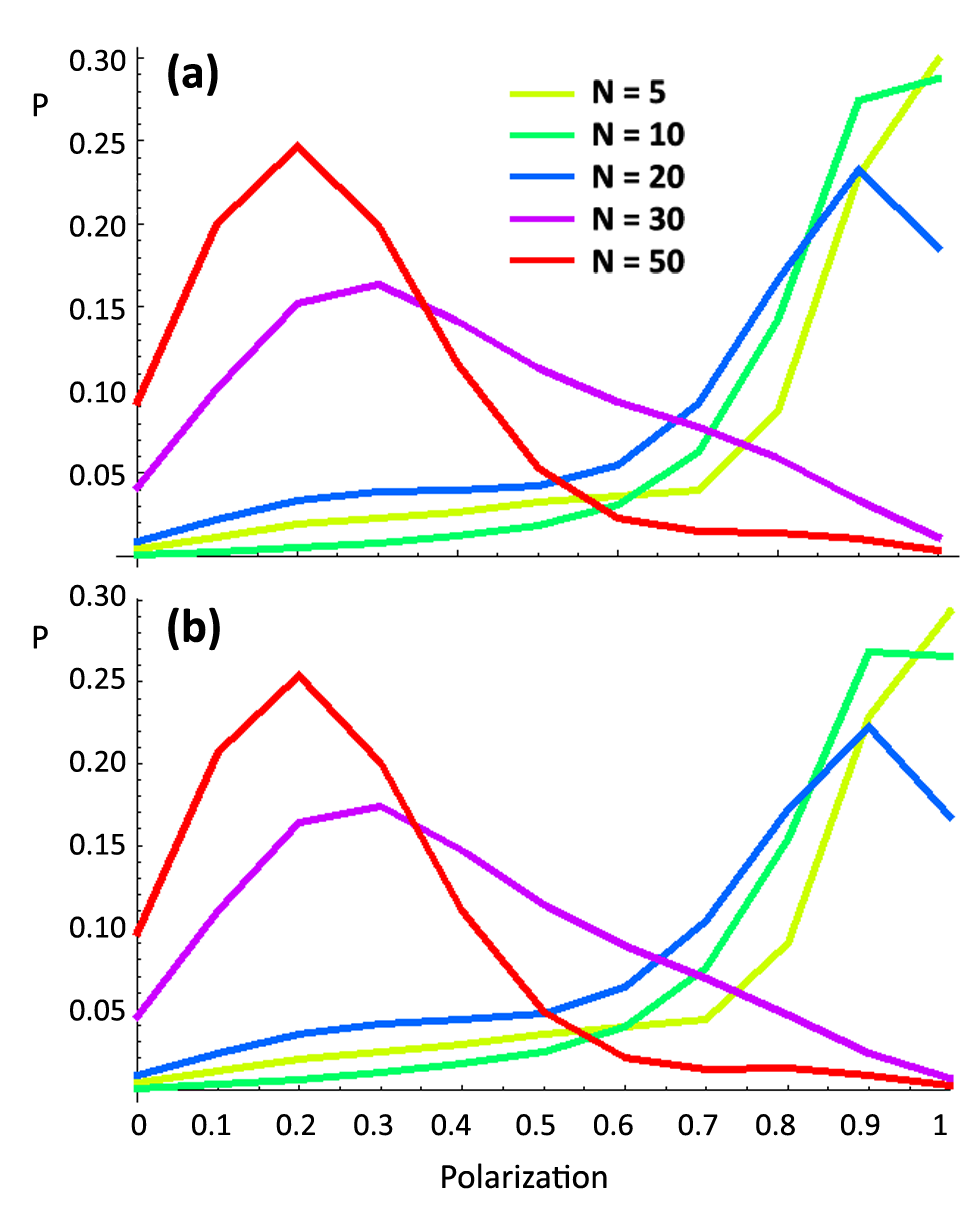

Supplement: Figure S1 — Polarization distributions for Experiment 3, using alternate measures of polarization. These figures display the same data as Figure 5 in the main text, but calculated using an alternate measure of polarization that eliminates potential effects of the curved testing tank walls (a) and without excluding fish that had left the group on excursions (b). All comparisons between the distributions for different group sizes were qualitatively identical to those for the original data, presented in the main text. (TIF) [file pone.0048865.s001.tif]

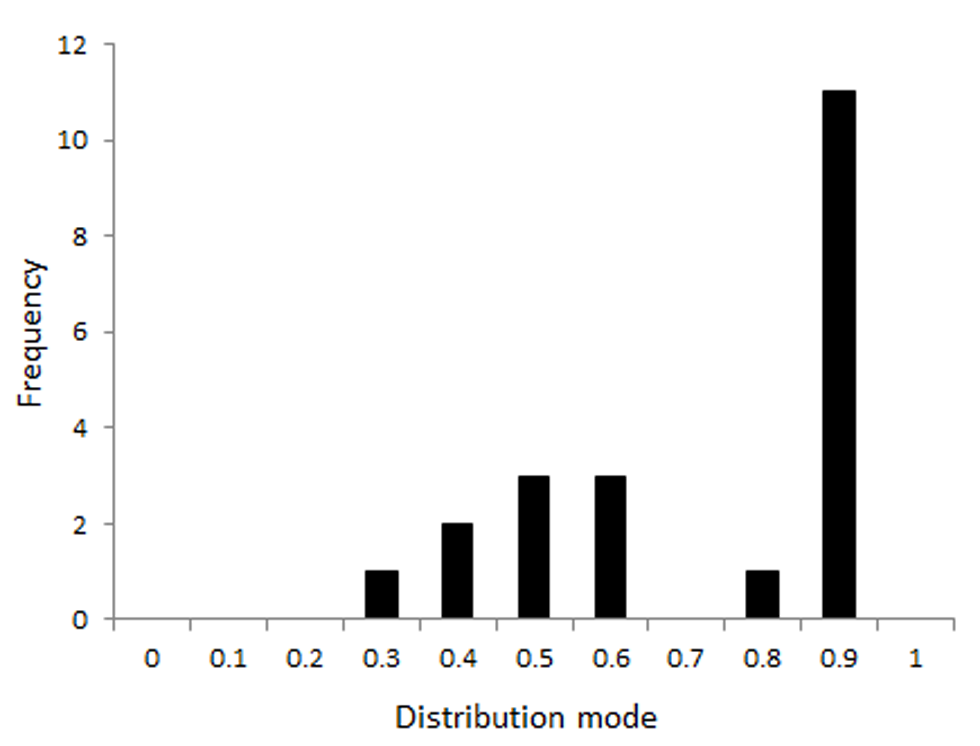

Supplement: Figure S2 — Binned modes of unimodal polarization distributions in Experiment 1. The largest number of unimodal distributions occurred on the first day of exposure to the testing environment (8 of 21) and represent sessions in which the group spent most of its time schooling (high polarization). (TIF) [file pone.0048865.s002.tif]

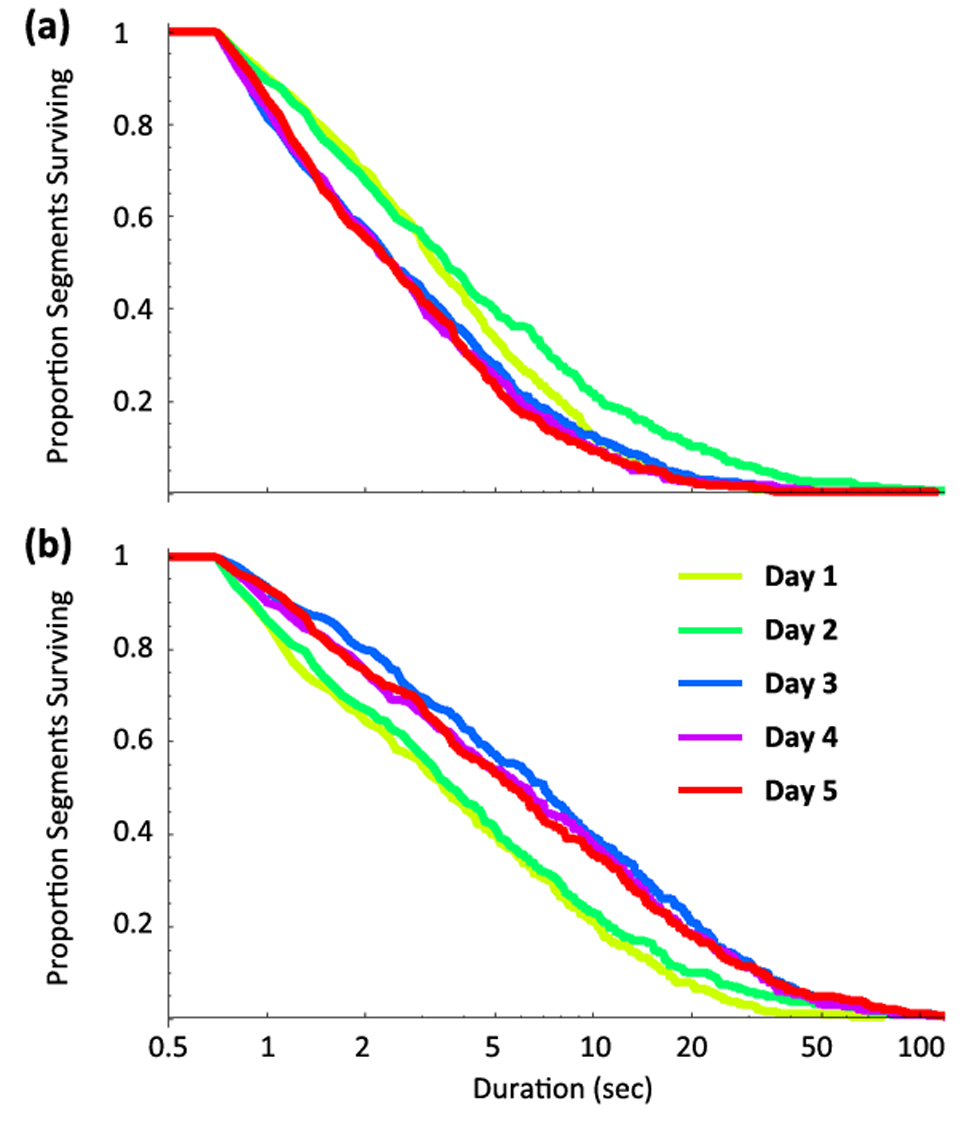

Supplement: Figure S3 — Survival curves for schooling (a) and shoaling (b) segments for Experiment 1. The curves show the proportion of segments of each mode that lasted longer than a given length of time. Mean schooling segment length was 4.7 sec and mean shoaling segment length was 9.4 sec. Data are plotted on a Log-linear axis. (TIF) [file pone.0048865.s003.tif]

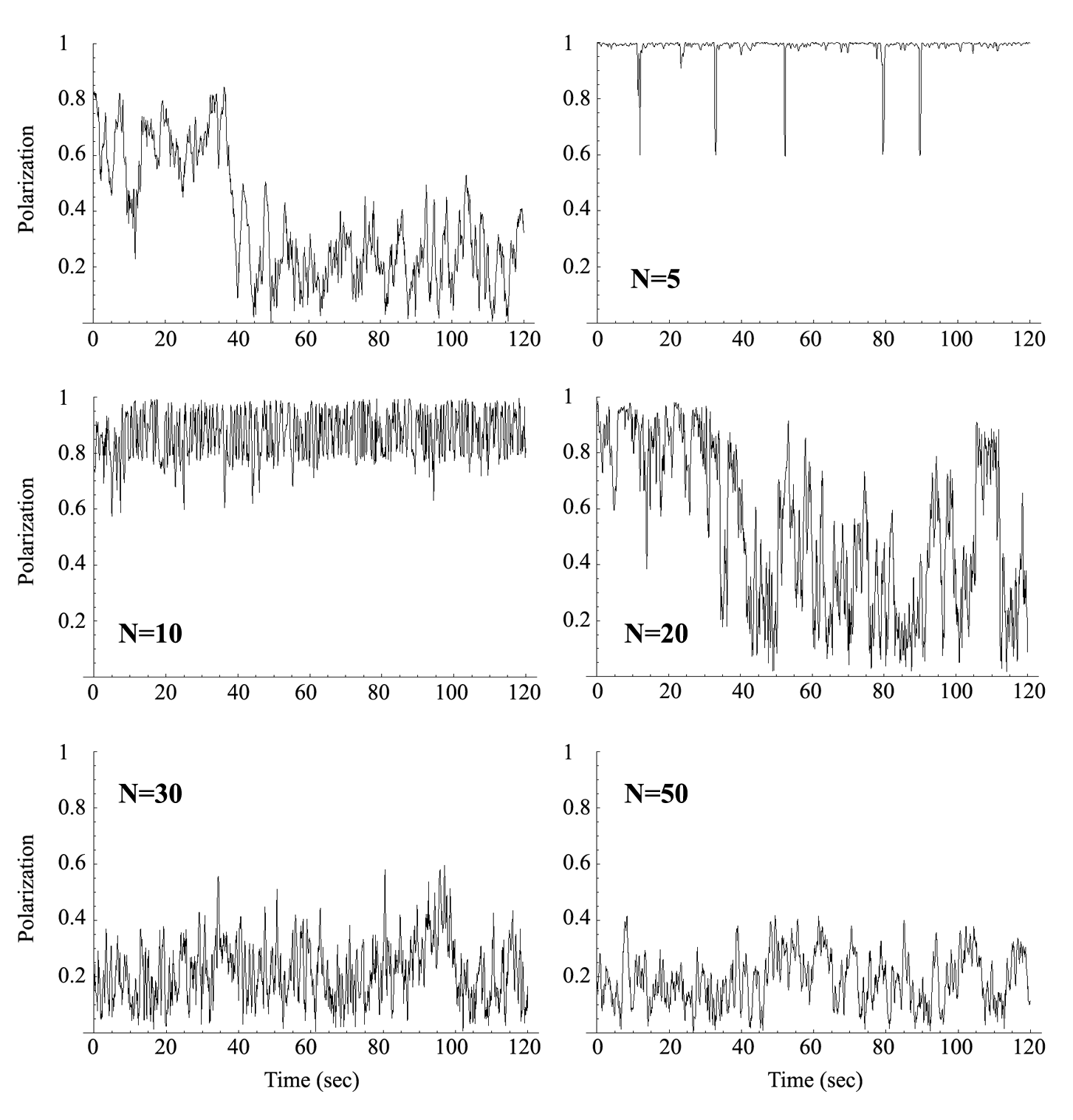

Supplement: Figure S4 — Sample time-series' of polarization. Each panel shows the polarization of a single group for the first 2 min of a sample session. The top left panel shows a transition from schooling (high polarization) to shoaling (low polarization) from Experiment 1 (N = 8). The other 5 panels show representative samples of the polarizations of groups of different sizes (N = 5, 10, 20, 30, and 50) from Experiment 3. (TIF) [file pone.0048865.s004.tif]

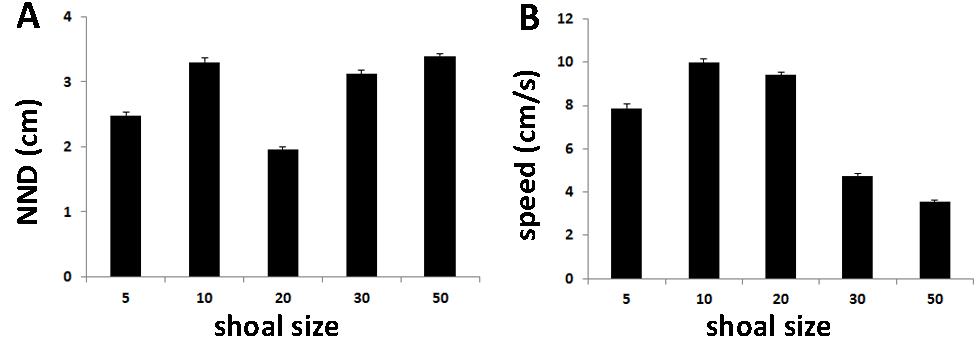

Supplement: Figure S5 — Mean NND (A) and mean speed (B) of zebrafish shoals of different sizes, from Experiment 3. The charts show the mean NND and mean speed over all frames of all groups at each shoal size. Error bars represent ± SEM. (TIF) [file pone.0048865.s005.tif]

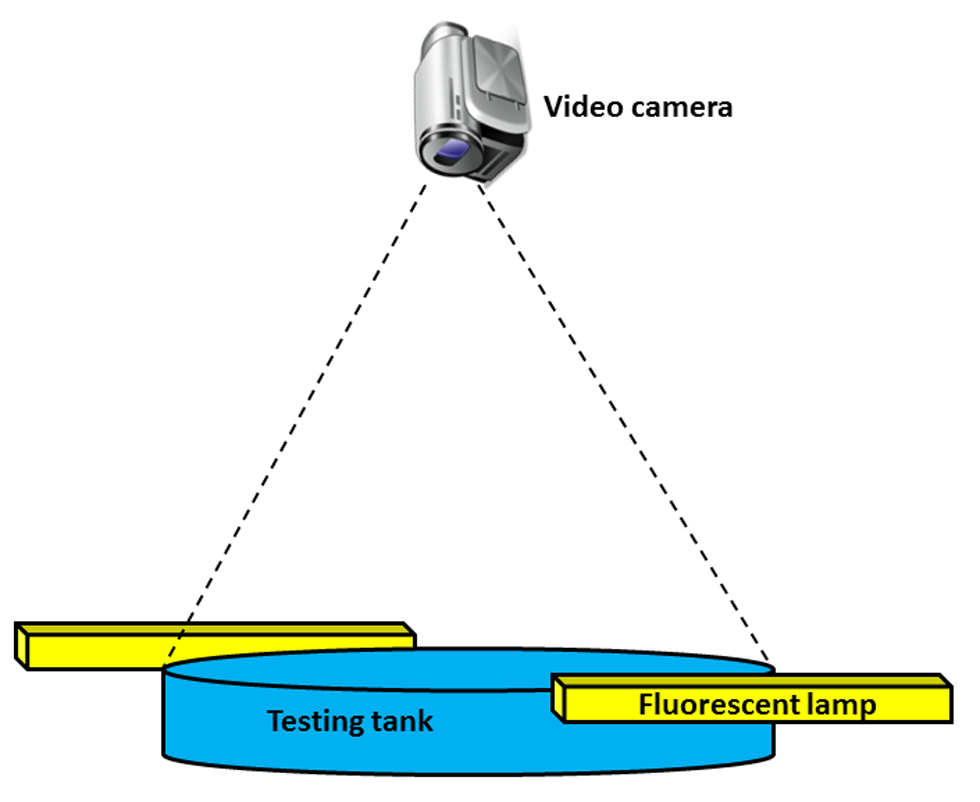

Supplement: Figure S6 — Diagram of the experimental setup. The camera was mounted on the ceiling such that the entire testing tank was visible in the frame. Two fluorescent lamps lit the tank from the sides, placed just above the lip of the tank to avoid glare in the video image. The tank was 91 cm in diameter and filled with water to a depth of 10 cm. Adapted from Miller, N. & Gerlai, R. (2012). Automated tracking of zebrafish shoals and the analysis of shoaling behavior. In A.V. Kalueff and A.M. Stewart (eds.) Zebrafish protocols for neurobehavioral research (New York: Humana Press) pp. 217–230. (TIF) [file pone.0048865.s006.tif]
