# Supplementary material for: From Schooling to Shoaling: Patterns of Collective Motion in Zebrafish (Danio rerio)
Source: PLoS One. 2012 Nov 14;7(11):e48865. doi: 10.1371/journal.pone.0048865 (PMC3498229; doi:10.1371/journal.pone.0048865)
Supplement: Table S1 — Comparisons of polarization distributions by day in Experiment 1. Summed distributions (shown in Figure 1) were compared between repeated exposures to the testing environment using a 2-sample Kolmogorov-Smirnov test. The top half of the table presents the test statistic values; the bottom half presents p-values. Non-significant p-values are shaded. (PDF) [file pone.0048865.s007.pdf]

Day	1	2	3	4	5
**1**	--	0.024	0.269	0.229	0.368
**2**	1	--	0.249	0.210	0.348
**3**	< 0.0001	< 0.0001	--	0.040	0.100
**4**	< 0.0001	< 0.0001	0.965	--	0.141
**5**	< 0.0001	< 0.0001	0.091	0.005	--
