# Supplementary material for: From Schooling to Shoaling: Patterns of Collective Motion in Zebrafish (Danio rerio)
Source: PLoS One. 2012 Nov 14;7(11):e48865. doi: 10.1371/journal.pone.0048865 (PMC3498229; doi:10.1371/journal.pone.0048865)
Supplement: Table S2 — Comparisons of polarization distributions by hour in Experiment 2. Summed distributions (shown in Figure 2) were compared between hours of the session using a 2-sample Kolmogorov-Smirnov test. The top half of the table presents the test statistic values; the bottom half presents p-values. Non-significant p-values are shaded. (PDF) [file pone.0048865.s008.pdf]

Hour	1	2	3	4
**1**	--	0.492	0.524	0.555
**2**	< 0.0001	--	0.055	0.088
**3**	< 0.0001	0.990	--	0.035
**4**	< 0.0001	0.692	1	--
