# Supplementary material for: From Schooling to Shoaling: Patterns of Collective Motion in Zebrafish (Danio rerio)
Source: PLoS One. 2012 Nov 14;7(11):e48865. doi: 10.1371/journal.pone.0048865 (PMC3498229; doi:10.1371/journal.pone.0048865)
Supplement: Table S3 — Schooling and shoaling segment lengths by day in Experiment 1. Mean durations of schooling and shoaling segments for each day of Experiment 1. Mean segment lengths ± standard deviations are given in seconds. (PDF) [file pone.0048865.s009.pdf]

Day	1	2	3	4	5
Schooling	4.51 ±	7.40 ±	4.15 ±	3.75 ±	3.67 ±
Shoaling	5.73 ±	7.78 ±	11.62 ±	10.51 ±	11.35 ±
