# Supplementary material for: From Schooling to Shoaling: Patterns of Collective Motion in Zebrafish (Danio rerio)
Source: PLoS One. 2012 Nov 14;7(11):e48865. doi: 10.1371/journal.pone.0048865 (PMC3498229; doi:10.1371/journal.pone.0048865)
Supplement: Table S4 — Comparisons of polarization distributions by group size in Experiment 3. Summed distributions (shown in Figure 5) were compared using a 2-sample Kolmogorov-Smirnov test. The top half of the table presents the test statistic values; the bottom half presents p-values. (PDF) [file pone.0048865.s010.pdf]

**Group Size**	**5**	**10**	**20**	**30**	**50**
**5**	--	0.181	0.412	0.721	0.790
**10**	< 0.0001	--	0.295	0.785	0.889
**20**	< 0.0001	< 0.0001	--	0.612	0.758
**30**	< 0.0001	< 0.0001	< 0.0001	--	0.314
**50**	< 0.0001	< 0.0001	< 0.0001	< 0.0001	--
